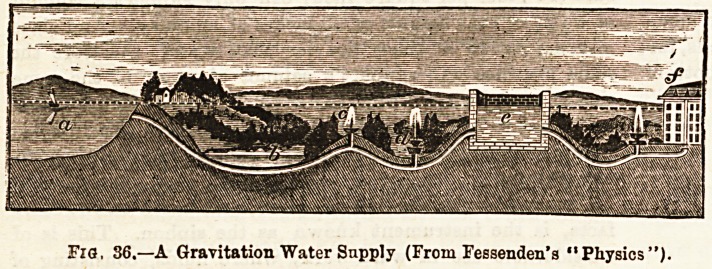# "The Hospital" Nursing Mirror

**Published:** 1896-08-01

**Authors:** 


### "The Hospital" Nursing Mirror.

Being the Extra Nursing Supplement of "The Hospital" Newspaper.

[Contributions for this Supplement should be addressed to the Editor, The Hospital, 428, Strand, London, W.C., and should have the word "Nursing" plainly written in left-hand top corner of the envelope.]

### News from the Nursing World.

### THE RIVAL BABIES.

It will be remembered that in the account we published of the Royal wedding last week attention was drawn to a very attractive baby who made his appearance on the wall of Marlborough House to the left of the garden entrance. Many conjectures were made as to the identity of this baby, so we may state that it was the little son of Sir Francis and Lady Knollys, a delightful little boy and a general favourite. Many of the crowd thought that little Knollys was none other than the Prince Edward of York. Prince Edward of York was also a spectator of the procession, but he had taken up his station on the other side of the road at a French casement in St. James's Palace, from which he looked down on the crowd below. He was detected by some of the crowd, and, like the baby at Marlborough House, attracted a good deal of attention. Both babies were cheered, and Prince Edward won the hearts of the crowd by the bewitching bows which he made in response to the cheers which greeted his appearance. No one who witnessed the conduct of the two babies can doubt that they thoroughly enjoyed the attentions which were lavished upon them. Indeed, Prince Edward of York enjoyed it so much that he was reluctant to leave the window, a fact noticed by the crowd, who cheered him again and again after his disappearance, which brought the baby once more to the window, from which he again bowed his acknowledgments. Few Royal weddings have been more popular than that of last Wednesday week, and every sort and condition of men have sent verses, cards, and tributes to the bride and bridegroom, to the no small embarrassment, we imagine, of the recipients, who must have been overwhelmed by these attentions from every part of the country.

### THE VICTORIA HOSPITAL FOR CHILDREN.

The Princess Louise, Marchioness of Lorne, patron of the Victoria Hospital for Children, and the Earl and Countess Cadogan, president and vice-president of the hospital, have given their patronage and support to the grand Imperial fête and fancy fair to be held in June next in aid of the endowment of the hospital. The Royal Botanical Gardens will probably be the scene of the fête, and arrangements are being made with Madame Tussaud's for the reproduction of the scene representing the coronation of the Queen in 1837. Another feature of the fête will be the erection of a large Imperial crown, which will hold the same position as the wedding cake at the silver fête.

### THE GRANDCHILDREN OF OUR QUEEN.

It may interest nurses to know that *The Gentlewoman* is now publishing portraits of the 56 grandchildren and great-grandchildren of the Queen in weekly sets. The Royal Family are especially beloved by nurses for the kindness and sympathy they have shown towards them, and no more loyal class is to be found than that which is occupied in tending the sick. Portraits of members of the Royal Family are much appreciated, and we seldom enter a nurse's room without finding an instance of this.

### ASYLUM NURSES.

At the annual meeting of the British Medico-Psychological Association a paper was read by Dr. Turnbull, Medical Superintendent of the Fife County Asylum, on a very successful experiment which had been made in his institution in the direction of placing the male sick room of the asylum under the care of female nurses. The result was in every way satisfactory. Dr. Savage, in the discussion which followed, said that the employment of female nurses rather than male was preferable in most cases of advanced general paralysis, and many cases of senile dementia. In private practice men suffering from these affections opposed most strongly the exercise of any control by anyone drawn from the class to which male attendants generally belonged, and it excited them in an extreme degree. Old patients who could not be controlled by men submitted to female nurses as nicely as possible. The more highly educated and refined the lady nurses, the more amenable were the patients.

### HASTY CONCLUSIONS.

The death of Nurse Hilda Smith at the Children's Hospital, Pendlebury, was a very painful affair, resulting from a dose of chloroform, taken when the nurse was labouring under distress of mind. Great sympathy must be felt with the friends of the nurse, and also with the managers of the institution. The latter, as is often the case under similar circumstances, have been hastily blamed for overworking their nurses. We are surprised that so far we have not seen this contradicted. The work at a children's hospital is well known to be lighter than where adults are admitted, and we have frequently known Pendlebury recommended as a suitable introduction to nursing duties by reason of the lightness of the duties and comforts provided for the nurses.

### DISTRICT NURSING.

All over the country, district nursing is being established with quite astonishing rapidity. Only during the last few days we notice that at Brighton, Orlestone, Somersham, Thrapston, Bathgate, Buckhurst Hill, Salehurst, and Romford district nursing has just been or is about to be established. Several first annual reports of district nursing associations have recently been issued, and they all show a record of success, and testify to the appreciation expressed in the various districts.

### NURSES' QUARTERS.

An extraordinary statement was made at a recent meeting of the Board of Guardians of Mallow, in Ireland. This was that the sleeping accommodation of two of the nurses acted as a passage between one part of the Union infirmary to another, one of the nurses being a night nurse, and therefore using her room during the daytime. It was not reported that any alteration of the system was arranged at the meeting.

### NURSES AND TRAMPS.

The nursing arrangements at the Totnes Union Infirmary are in a very unsettled state at present. The three nurses employed are supplied by the Workhouse Nursing Association, and all three last appointed have resigned, owing to the rules providing that the admission of tramps into the workhouse and their subsequent bathing should be superintended by the nurses. The Guardians have modified the rule by exempting the head nurse from this duty, but require it to be followed by her assistants. The last application to the association for nurses was responded to, but the secretary expressed the hope that the rule would be rescinded. At the meeting of the Guardians, at which the matter was discussed, a general feeling of apprehension appeared to prevail that if the Board severed its connection with the association (as some of the Guardians wished) the supply of nurses would fail altogether. At a subsequent meeting a letter from the Workhouse Nursing Association was read, in which it was stated that the association feared they would have to withdraw the nurses if no alteration was made. The guardians wisely, we think, came to the conclusion that in the future the nurses must not be required to receive the paupers on admission to the workhouse.

### MUSIC IN THE WARDS.

The ward which possesses a piano or harmonium holds an envied position, and now two wards at the Newcastle Infirmary are the fortunate possessors of a piano each. It is the custom to hold concerts in the wards of the infirmary, and the welcome gifts are especially useful. The donors are the members of the Co-operative Wholesale Society of Newcastle, and the makers, Messrs. Pohlman and Son, of Halifax, have contributed by providing the instruments at cost price. The inscription run, "Presented to the Royal Infirmary by the workmen of Tyneside and district." A concert was held to celebrate the presentation, which was much enjoyed and largely attended by those interested.

### NURSING AT WOLVERHAMPTON.

The Queen Victoria Nursing Institute at Wolverhampton has an encouragingly successful year's work to report. From a temporary home the institute has been transferred to suitable permanent quarters, for the furnishing of which generous help was forthcoming from a few kind friends. Six paying patients have been received into the home, this being a branch of the work which the committee hope will increase. The past year was begun with a balance in hand of £419, of which £400 was transferred to the building fund of the new home, and the income for the year exceeded the expenditure by £294, without any subscriptions. The report especially made mention of the never-ceasing interest in the welfare of the institution taken by Miss Marianne Lowe, its hon. secretary, and of the "energy and devotion" of the lady superintendent, Miss Loveys. The name of this institute is a little misleading, naturally being now and again confused with the Q.V.J.N.I., of which there is no branch at Wolverhampton. The committee would, we think, be well advised to make some alteration, for the better avoidance of mistakes in future.

### NURSES' QUARTERS AT HALIFAX.

At the New Royal Halifax Infirmary, which was opened by the Duke and Duchess of York on the 25th ultimo, the accommodation for the nurses is very good, although separate bedrooms do not seem to have been provided for all the probationers. The nurses' sitting-rooms and library are extremely pleasant apartments.

### "NURSING NOTES."

We beg to call the attention of nurses generally, and especially of district nurses, to the special Queen's Nurses' Number of *Nursing Notes* for August, which contains a full account of the ceremony at Windsor Castle, by Miss Dunn, the general superintendent of the Irish branch, and many items which cannot fail to prove of special interest not only to Queen's nurses, but to all who are interested in nursing matters.

### A FETE AT CANTERBURY.

A most attractive and successful fête was held at Canterbury, in aid of the Kent and Canterbury Hospital. The weather was glorious, and numbers of people hastened to Ersham House, in the grounds of which the fête was held. Part of the entertainment was a horticultural show, whilst a display by some of the men from the Cavalry Depôt who had assisted at the Military Tournament was much appreciated. Other soldiers gave an exhibition of tent-pegging, lemon-cutting, &c. A musical ride and fire brigade display were especially popular. Numbers of ladies and gentlemen from the locality lent their services.

### SHORT ITEMS.

The nurses' home at the Liverpool Workhouse is to be extended at the cost of £7,500, and at Reading the nurses' accommodation at the union infirmary is also to be added to.—The Bedford Board of Guardians recently engaged a nurse for their infirmary. Of the four selected candidates not one was a trained nurse, according to the statement of a medical man present when the engagement was made.—Good work has been done through the Sheffield Nurses' Home and Training Institute. The institution has met with much encouragement locally.—Nurse F. Walker, of the Greenwich Union Infirmary, has been presented by her patients with a framed and illuminated address in token of her kindness to them.—The good people at St. Agnes are highly pleased with the result of the first year's work of their district nursing association. —At Hucknall Torkard, nineteen candidates received certificates after passing an examination held in connection with the County Council nursing lectures.

### Hygiene: For Nurses.

By John Glaister, M.D., F.F.P.S.G., D.P.H.Camb., Professor of Forensic Medicine and Public Health, St. Mungo's College, Glasgow, &c.

### XVII.—HOW WATER REACHES OUR HOMES—THE PUMP—GRAVITATION—THE SIPHON—THE HYDROMETER.

The source of a water supply largely determines the mode by which water reaches the homes of the users. In wells, for example, there is a collection of water lying at the bottom

Fig. 32. The Lifting Pump.

Fig. 33.

of a shaft, more or less deep, which, before it can be used, must be lifted. This is usually accomplished either by a windlass, or a hand-rope and bucket, or by a pump. Of the former not a word more need be said, but of the latter some explanation must be offered.

Everyone is acquainted with the fact that, if an ordinary glass syringe is dipped into water and the piston pulled out, water will rise in the barrel. This is due to the difference of atmospheric pressure within the barrel at the moment of drawing the piston, and that on the surface of the water in the vessel from which it is drawn. The pressure being greater on the latter than in the former, the water is forced into the barrel, following the piston. In this simple experiment several of the points in the action of a pump are demonstrated. To Galileo and Torricelli we are indebted for our knowledge of pump-action. The ordinary pump is called the lifting-pump, or the suction-pump. Figs. 32 and 33 will assist in the comprehension of the following explanation of the action of a pump. A pump consists of these essential parts, viz.: (1) The piston which runs in the pump-barrel; (2) the pump-barrel, within which the accurately fitting piston is moved up and down by the pump-handle, and from which the spout opens; (3) the pump-tube, which extends from the bottom of the pump-barrel to some depth in the water of the well; (4) the pump-handle which moves the piston; and (5) certain valves, one in the piston, and one in the pump-tube. When the piston is raised the piston-valve is closed, because of the pressure of water above it, and water is forced out of the spout; at the same time the pump-tube valve is opened and water is forced into the barrel, as in the syringe, and for the same reason. When the piston has reached the highest point of the upstroke, it is now made to descend in the barrel, when the piston-tube valve opens and the pump-tube valve closes. By this alternate up and down movement of the piston by the pump-handle, therefore, the water is raised from the well into the pump, and from the pump-spout, vessels may be filled. Indeed, the operation is accomplished in less time than it takes to describe it. But the lifting pump can only raise water from a limited depth, which, in theory, is about 34 feet, but in practice from 25 to 30 feet. This limit is due to the fact that ordinary atmospheric pressure (14·7 lbs. per square inch) can only support a column of water of that height, or 30 inches of mercury. And since mercury is 13·59 times heavier than water, therefore the same pressure will support a column of water of the same size to a height equal to 30 in. × 13·59, viz., 34 feet. When water has to be raised from greater depths than this different pumping apparatus must be used, which, however, is beyond our purview.

Connected with the flow of water, and based on the same facts, is the instrument known as the siphon. This is of considerable use in ward work, where fluids, consisting of layers of different densities require to be separated, or a fluid from a deposit, or in transferring antiseptic fluids from reservoirs to the operating-table. Besides, it is used in the treatment of certain gastric diseases, and in emptying the contents of the stomach in cases of poisoning. Fig. 34 shows a glass siphon which is always ready for use. To set the ordinary siphon in operation all that is necessary, provided the nature of the fluid permits, is to suck, or by the use of a syringe, to exhaust the air out of the long limb of the siphon after placing the shorter limb in the vessel, when the fluid will run in a continuous stream until the level of the liquid in the vessel reaches the extremity of the tube. The form of the siphon shown permits this to be done very easily.

Another instrument of use in hospital and private nursing is the hydrometer, which is intended to show the specific gravity of fluids. Fig. 35 shows one form of the instrument. The ordinary urinometer—or hydrometer graduated for this fluid—is composed of a bulb containing mercury or small shot, a second bulb containing air, and a stem, in which is enclosed a graduated scale. The sp. gr. of any liquid is the weight of a unit volume of it compared with an equal volume of a standard fluid. Water at 4 deg. C., or 39·2 deg. Fahr., is the standard fluid, and its sp. gr. is 1, or, as it is usually

Fig. 34.—Syphon-tube.

Fig. 35.—Urinometer.

expressed, 1,000. In using the instrument, a cylindrical vessel is filled two-thirds full of the liquid, to be tested, into which the hydrometer is placed. The depth at which it will float depends on the weight or density of the fluid, and the point on the stem at which it becomes stationary, and which is at the level of the surface of the fluid is then noted, and the figure corresponding to that point written down. This registers the sp. gr. of the fluid tested. Special instruments are made for special fluids, viz., the urinometer for urine, the lactometer for milk, the saccharometer for sugary liquids, and the oleometer for oils; and they are graduated within the limits of gravity known to be possessed by these different classes of fluid substances. In addition to being used for testing the specific gravity of urine it is likewise used for fluids from tumours, or from the natural cavities of the body. The lactometer, used for taking the specific

Fig. 36.—A Gravitation Water Supply (From Fessenden's "Physics").

gravity of milk, is worse than useless as indicative of the purity of the sample, for reasons which space forbids us to enter upon.

To return, however, to water supply to the house. When populous places are to be supplied from natural or artificial collections of water, the relative heights of the source and the area of distribution become of importance. Water will run down hill whether in an open channel or in a pipe, owing to the law of gravity; hence such supplies are often designated gravitation supplies. To be of the greatest service the level of the source must be some feet higher, at least, than the highest point of distribution, else the water will not rise to houses on this level. Sometimes, however, this cannot be avoided, and in such cases auxiliary pumping-stations must be erected to supply the higher levels. Fig. 36 illustrates in section a gravitation supply. From the lake, *a*, the dotted line being the level, the water flows under the river, *b*, into a reservoir, *e*, and from thence to the houses, *f*. Should, for any reason, the level of the water in the lake fall much, the top flats of the houses, *f*, would not be supplied because the pressure would be insufficient, since the fall would be equally experienced in the reservoir, *e*.

From such gravitation supplies the supply to houses within the area of distribution is either continuous or constant, or intermittent, *i.e.*, either on a tap at any time of the night or day, or for periodic intervals only, in the course of the day. Adequacy of supply is the chief determining cause of this. Where water is abundant constant supply prevails, where barely adequate, the intermittent. In the latter case it is necessary to erect house cisterns wherein to store water against the time when the main supply is cut off. This entails risks of pollution, with attendant harmful results to the consumers. A supply of water ought, therefore, to be continuous in action, plentiful in amount, and it ought to be easy to obtain by every householder, in order to contribute to cleanliness, both public and domestic.

The amount of water to be supplied per head of population per day is necessarily regulated by the abundance of supply. But there is a certain minimum which is necessary. The average adult needs for nutrition from 70 to 100 oz. daily. Each soldier in barracks is allowed 15 gallons daily. From 12 to 15 gallons per day may, therefore, be considered a fair minimum. The supply per individual may be apportioned as follows, viz.:—Dietetic: (1) cooking; (2) drink; Sanitary: (3) personal ablution, (4) cleansing of clothing, (5) as a vehicle for sewage.

In towns the amount per head per day ranges from 20 to 300 gallons, many towns in England giving the former, and New York the latter amount. These amounts cover a variety of purposes, viz., personal and domestic cleanliness, including baths and closets, animal nutrition and cleansing, municipal and manufacturing uses. The citizen of London receives, on the average, 28 gallons daily; of Liverpool, 30; of Paris, 31; of Edinburgh, 35; of Glasgow, 50; and of Rome, 100. These are typical city supplies.

### New York City Training School for Nurses, Blackwell's Island.

The annual report, read at the twenty-first annual meeting of this school by Miss Darche, its superintendent, is an interesting one, showing that the school has lately made advancement in several directions. In the first place, during the past year the school has been made responsible for the nursing of Fordham Hospital, an institution where previously confusion had reigned supreme. The plan of management introduced is similar to that in force in the Gouverneur and Harlem Hospitals, with which institutions the school has been for some years connected. A graduate of the school was appointed supervising nurse and matron, a staff of nurses placed in the wards, and much hard pioneering work gone through. The results so far have been excellent. Another improvement effected has been the appointment of an advisory board for the school, an action by which the commissioners have secured to the school a connexion which strengthens and supports its administration, and the weight of responsibility hitherto borne by the superintendent has been lessened by the share taken in it by the ladies who have been appointed advisors. An advance has been made by the appointment of graduate head-nurses, made possible by the liberal grant to the school of ten salaries of each 360 dols. per annum. Six salaries were allowed for providing head nurses for the City Hospital, one for the head night nurse at Maternity Hospital, and one each for the Fordham, Harlem, and Gouverneur Hospital. It is intended to give each graduate of the school in turn, who is possessed of the requisite managing ability, the opportunity to fill for a definite period these advanced posts of responsibility, and the Commissioners have decided that a post-graduate course of six months shall be established as one of the features of the school, a special diploma, certifying to managing powers, being granted at the expiration of this training. The Male Training School has been steadily improving in tone and character. The applicants have increased in numbers, and these have come from a more reliable class of men, so that failures and dismissals have been fewer; they have, indeed, been reduced 50 per cent. The school numbers twenty-five members, and eight young men will graduate this year. The School Registry grows in favour with medical men and the public, and the demand for nurses is increasing. The registry is managed on the co-operative plan, and the fees are sufficient to cover the expenses incurred in its management. During the past year there have been 475 applications for admission into the school; forty-eight nurses have been admitted on probation, eight have dropped from various reasons, and twenty-one graduate this year.

### The Junius S. Morgan Benevolent Fund.

We are requested to acknowledged in our columns donations towards the above fund of sums varying from 1s. to 2s. 6d. from Nurses J. H., G. L., E. M., E. O. H., and C. G., all of the Sunderland Institution for Trained Nurses.

### Nurses in 1896—Their Quarters, Hours, and Food.

### I.—INTRODUCTORY.

A GREAT deal of interest is taken by the general public in all matters connected with nursing and nurses. This interest has been too great for the good of the nursing profession in the opinion of some people, who think its members are none the better for the attention which has been so strongly directed to them of late. Seeing, however, that so much comment, adverse and otherwise, has been made upon the hours, feeding, and accommodation provided for nurses in the present day, we propose to give the actual facts, and to consider the whole subject from data obtained direct from the most representative hospitals and institutions. With this view it is proposed in a series of articles to deal with certain London hospitals and infirmaries, first taking those with separate nursing homes, and then those not possessing such accommodation.

In collecting together this information it has been remarkable to notice how widely opinions differ amongst matrons and hospital authorities as to the requirements of their nurses, and this on many essential points. Whilst, for instance, at one well-known London hospital every nurse in the building is off duty for four hours each day, and the actual hours on duty, including sufficient time for two meals, amount to between nine and ten hours, at another the nurses are only off duty on three days in the week, and the ordinary working day averages thirteen hours and a half. It must be conceded at once that the time off duty must of necessity vary at different hospitals, and that the disproportion in the matter of hours is not always so great as appears at first sight. For instance, at one institution the nurses may be hard at work during their nine or ten hours in the wards, while at another, though nominally on duty for thirteen or fourteen hours, during three or four of these they may be quietly occupied with work of their own. But it is undeniable that in every hospital, for a definite portion of each working day, the nurses ought to be entirely away from the wards, and at liberty to go out for fresh air and exercise, or to lie down and rest tired feet.

With regard to accommodation there is also much variation between the different hospitals. Indeed, taken as a whole, some of the modern poor law infirmaries, with their entirely separate nurses' homes and comfortably furnished sitting-rooms, put several of the voluntary hospitals in London to shame. At these hospitals it is still possible to find bedrooms of a very inadequate size shared by two nurses, with only one washhand-stand and dressing-table between them. Most of the present nurses' quarters in the London hospitals date from a time when cubicles were held in high favour, and were supposed to possess certain superiorities in the way of ventilation over small separate rooms. This idea has exploded with time, and the right of every nurse to a separate room, and that undisturbed rest which it is impossible to obtain in any cubicle, has been clearly demonstrated by those best able to judge. In nursing homes of the future the cubicle will no longer find a place. Many hospitals of to-day are so badly planned that the ward sisters are provided with bed-rooms as well as sitting-rooms off their ward. These rooms in some cases are almost in the ward itself, being merely separated from it by a matchboard partition. Such an arrangement must materially detract from the comfort, health, and sleep of the unfortunate occupant. The desirability of ward sisters having their private sitting-rooms leading from their ward has been much debated, and varies with the class of hospital. In very large wards, where the sister has under her care as many beds as the matron of a small hospital, she undoubtedly needs some place of retirement where she may yet be within call if necessary. In small wards, where the sister holds the position of charge-nurse, she is actually needed in her ward during her whole time on duty, and so has no proper use for such a sitting-room.

There would seem to be very little fault to be found by the most captious critic on the score of the food provided at most hospitals for the nursing staff. It is usually ample in quantity, and the quality is as good as it is possible to provide in catering for a number of people. The difference lies in the manner of serving the food—a point which depends for the most part on the capacity of the matron of each institution. Where the matron is particular about clean table-linen and little niceties of serving, there other refinements prevail, and the difference is very noticeable. In some few hospitals there still lingers the utterly indefensible custom of allowing certain rations weekly to each nurse, *e.g.*, butter, sugar, tea, and bread for the morning lunch and afternoon tea, which meals they are permitted to take in the wards, or at best in the ward kitchen. For nurses to be allowed to keep butter and bread in a cupboard in a ward is unwholesome and nasty, and that such a thing should be possible in these days of asepticism is quite astonishing. For night nurses it is difficult to see how certain of the meals can be taken anywhere but in the ward kitchen. Where this is unavoidable the food should be served out freshly each night, and never be eaten in the ward itself. In modern hospitals there seems no reason whatever why afternoon tea and lunch should not be prepared and eaten in the nurses' dining-room. At the best-managed hospitals this plan is followed, as will be seen when the institutions themselves are dealt with.

In considering the conditions of nurses' work in the present day we can never forget the very large part examinations and the attainment of theoretical knowledge play in the training of a modern nurse. A very considerable portion of a probationer's off-duty time is devoted to working up her lectures, attendance at which is compulsory for the most part. Besides, her ultimate success in her profession must largely depend upon the certificate which she ultimately gains, so that she necessarily has little spare time for recreation, but is forced to cram her mind with a sufficient knowledge of anatomy and physiology to carry her through her final examination. It is the combined strain of mental work and much hard physical labour which renders it essential that each probationer shall have a definite number of hours off duty each day and so many days and weeks' holiday in the year. The amount of yearly holiday customary has expanded a good deal of recent years, and three weeks instead of a fortnight for probationers, and a month or five weeks for sisters and head nurses, is usually now given. Every nurse should have a month's holiday in each year, and this is the end towards which those who are most interested in the welfare of nurses are aiming. One day a month, and a half-day a week, are given in some instances, and in one institution, at least, a whole day once a week is what is hoped for in the future. One point comes strongly out in comparing the hours at the different hospitals, and that is that as a rule nurses do not have a sufficiently long night. Eight hours in bed is the proper amount for women engaged in such work as nursing, work which under the most favourable conditions cannot fail to try the nerves of the strongest; yet it will be seen that in some instances seven hours, and even less, are considered sufficient.

This brief summary of the points which will be touched upon in these articles would be incomplete without mentioning the question of wages. It has been said that in time nurses will all come to pay for their training instead of being paid for their services as at present, and in some institutions it is now the custom not to pay probationers during their first year. At present the pay is very much the same everywhere—probationers £12, £18, and £20 in their first, second, and third years' respectively. Board and lodging and a certain amount of uniform and washing are included, it being much more usual to provide washing of late years.

The usual period for which a probationer signs on entering a hospital is now three years, but in some instances it is unwarrantably extended to four years. Certificates are given at the end of two or three years' training according to circumstances.

### The Royal British Nurses' Association.

### WAS THE LETTER REGISTERED?

In our report of the annual meeting of the R.B.N.A. last week it is stated that Miss Breay's resolution was "ruled out of order by the chairman on the ground that notice had not been properly given, the resolution not having been sent in a registered letter." Miss Breay states this is absolutely correct, but she "stated at the meeting that the letter was registered, and that she held in her hand the official receipt of its registration, which she handed to the chairman." She omits, however, to add that the chairman said, after examining the document, "the receipt was merely one for an express message, and the letter was not registered." He therefore adhered to his rule that the terms of the bye-law had not been carried out. Miss Breay being dissatisfied with this decision on the part of the chairman, communicated with the Postmaster-General, and received the following reply, dated the General Post Office, July 27th, 1896: "In reply to your communications of the 11th and 22nd inst., I am directed to inform you that the letter to which you refer was duly delivered as a registered express letter, and its receipt was acknowledged by 'E. G. E. Guiseppi' at 2.50 p.m., June 30th, whose signature acknowledging the delivery is now in the possession of the department."

### Presentations.

There was a garden party at the Nurses' Home of the Birmingham Infirmary on Tuesday afternoon, July 14th, to say farewell to Sister Marion Foggett, who leaves to take charge of the New Workhouse Infirmary, Walsall. Sister Foggett joined the nursing staff as a probationer in October, 1889, completed her three years' training with the greatest credit, and was appointed sister of a medical, and later of a surgical ward. In September, 1894, she was appointed night superintendent. After tea in the nurses' sitting-rooms, Sister Foggett was presented with a very handsome gold watch by her fellow nurses, as a mark of their esteem and affection. The presentation, by request of the sisters and nurses, was made by the Matron, and Sister Foggett briefly returned thanks. Music and tennis followed, and a very pleasant afternoon was spent.

### Parade of Queen's Nurses.

We are requested to state that photographs of the "Parade of Queen's Nurses" at Windsor on July 2nd last, with the Queen's carriage in the centre of the square, can be procured at 5s. 6d. each, or printed in platinotype at 8s. 3d. each. Also the following groups: (Large size) Welsh Nurses, 5s. 6d.; Liverpool Nurses, 5s. 6d.; Glasgow Nurses, 5s. 6d.; (Cabinet size) Birmingham Nurses, 2s.; Chelsea Nurses, 2s.; Darlington Nurses, 2s.; Peterborough Nurses, 2s.; from Messrs. J. Russell and Sons, photographers, Windsor. Postal orders for amount should accompany each order.

### Ethics of Nursing.

The following is a report on the ethics and etiquette of nursing adopted by the Alumnæ Association of Nurses of the Johns Hopkins Hospital, at their annual meeting held June 4th, 1896.

### Introductory.

In formulating a code of ethics to be presented for the consideration of the Alumnæ Association the committee have studied carefully the national code of medical ethics. Valuable suggestions were also found in Dr. Flint's commentaries upon the national code of medical ethics, and also in the code of the Illinois Training School, Chicago. The work of the physician and of the trained nurse having so much in common from a moral standpoint, it of necessity follows that their laws of ethics must also be similar. It therefore seemed fitting to turn to their code for assistance, and thus benefit by their wider and longer experience in selecting a code of rules according to which their professional life should be governed. The code of ethics of the American Medical Association, adopted in 1847, nearly a half century ago, "has remained without any material additions and modifications." The committee appointed to draw it up based their code upon that of Thomas Percival, which was published in 1803, and from which the majority of the codes of ethics of different societies of the United States have been mainly taken. The national code of ethics of the medical profession has also been adopted by the state and local societies throughout the country. Bearing in mind that the Alumnæ Associations of Nurses look forward in the near future to the formation of both state and national associations, the committee, in drawing up the present code, have endeavoured to formulate it in such a manner that our alumnæ members will not be compelled to make any radical changes in it when called upon to conform to a national code of ethics.

The distinction between medical ethics and medical etiquette has been described by Dr. Austin Flint as follows: "The former rules have a moral weight, while etiquette, on the other hand, consists of the forms to be observed in professional intercourse, and are conventional." The committee have therefore appended certain rules of etiquette which may be regarded as to a certain extent supplementary to the laws of the code. In presenting their report they would especially insist that they have in no way aimed at originality, believing that in these matters mature experience, and not untried theories, would prove the safest guide.

The rules of conduct adapted to the many diverse circumstances attending the nursing of the sick constitute nursing ethics, and all such rules have a moral weight. Of the code of ethics adopted by the alumnæ it may be said that (1) it represents the views held by the majority of its members and is therefore binding on all; (2) it indicates the proper course to those whose moral perceptions may be insufficiently developed; (3) it may prove a safeguard against the bias of personal interests; (4) it is indispensable for the sake of reference whenever differences of opinion arise; (5) it thus contributes to the purity and dignity of the nursing profession.

The topics selected for the consideration of the alumnæ are as follows:—(1) The duty of the nurse to the physician; (2) the duty of the nurse to the patient; (3) the duty of the nurse to her school; (4) the duty of nurses to each other; (5) the duty of the nurse to the public; (6) the duty of the physician to the nurse; (7) the duty of the public to the nurse.

### The Duty of the Nurse to the Physician.

Sec. 1.—A nurse should strictly carry out the orders of the physician under whom she is working.

Sec. 2.—She should never discuss or criticise a physician with her patient or with the patient's friends. She should never express to them a preference for the services of any physician.

Sec. 3.—A nurse should always accord to a physician the proper amount of respect and consideration due to his higher professional position.

*(To be continued.)*

### On Certain Aspects of the Nursing Question as Seen in England and Germany.

By a Certificated Midwife.

### X.—A PATIENT'S EXPERIENCES IN GERMANY.

(*Concluded from page* lxxxiii.)

Into this abode of peace and quiet I was carried, and words fail to tell my thankfulness when I realised its stillness. Only those who have suffered from high fever will understand what the relief was to come away from cheerful home life with all its domestic noises; where the shutting of a door meant agony and a child's laugh seemed to set the brain on fire. "Here, if I die I shall die quietly," I said to myself. But in another moment I remembered the incessant worries to which (under orders) I had subjected the fever patients in England, and I shivered as I thought of what would be my fate. I quite expected a double dose of hot, heavy poultices, for I knew Germans were very "thorough" in their treatment, and when I saw a nurse standing by my bed I thought she was come to wash me with Condy, and inwardly I said, "Kismet." Outwardly, and with my tongue, I said nothing, partly because it was too great an effort, and partly because no one said anything to me. An icebag was placed on my head and another on my abdomen; a cup of hot milk and a saucer of broken ice was put on the little table where I could reach it. When it was found that milk was instantly rejected, I was not teased to take it or anything else. When my temperature reached a certain point Sister Röslie had orders to put me into a bath, which seemed to me to be ice cold, but I knew afterwards that it was lukewarm at first and cooled down when I was in; and there was no Condy. Once, but only once, I had two baths in the twenty-four hours. Sister Trinette and Sister Roslie lifted me out, wrapped me in a blanket, and laid me in bed, and I shall never forget the delicious sensation of relief from the whirl of pain and fever that these baths afforded me for an hour or two. And then I soon understood what was their object, and knew that when I was bad enough I should have another, so that there was always hope ahead of me. When I was taken out of my bath a large dose of dry quinine was given on my tongue, half a gramme I think it was, anyhow, enough to make me quite deaf. But this was the only medicine, as far as I remember, that was prescribed for me till I was out of the grasp of the demon fever. Being constantly sick at first, I entreated my doctor for some brandy; and I well remember his sarcastic smile as he answered, "Oh, you English! Brandy will do you no good." Later on he ordered me champagne, but in the tiniest doses, a teaspoonful at once; and I know that bottle, which held about half-a-pint, lasted me the whole time, for after twenty-one days, when the crisis was over, I had no more.

That three weeks, how long it seemed to me! No one was allowed to see me, no news was brought me of my friends. I lay like a log, with my brain in a whirl. Every thought and event of my past life roared and rushed before my inward vision. I never slept though I lay quite still in general. Twice or even three times a day I had a visit from the doctors. Dr. Essig was the house surgeon, and I did not like him for he would ask me questions. "Does your head ache, Fraulein? Have you balls of fire before your eyes?" and I would not try to answer what I thought such foolish questions, and then he would shake his head and go away. He was very young, and did not know any better than to talk. But when Dr. Teuffel came he would look at me with a cheerful smile, and say nothing. Truly silence is golden! His finger on my pulse, and the chart on which my temperature was noted, told him all he wanted to know; or if he had any questions he put them to my nurse. And so the days dragged on till one night I fell asleep soon after midnight and dreamt I was sitting on an iceberg, and then I woke up just as the clock was striking two, actually cold, and in a profuse perspiration. Good Sister Trinette, who was watching, for the crisis was expected, took away the icebags, and gave me a mouthful of meat jelly and my dose of champagne, and I was soon off to sleep again. And when my doctor came the next morning my temperature was normal, and there was nothing left but for me to get strong as soon as possible. The enemy had fled, and nature was the victor.

Henceforth my meals were my one joy, and I knew what it was to be an animal and hungry. But for another fortnight I was kept on broth and milk and red wine. At last a little *Kartoffelbrei* was allowed, but still I begged in vain for something solid. "You have cured me of the fever, *Herr doctor*," I pleaded, "but you will see me die of starvation." "What do you want, oh, unreasonable Englishwoman?" said he. "A slice of boiled chicken," was my modest answer, "and a bit of bread with it; something to bite, I beseech you." "Not for worlds," was the answer; "do you wish for a relapse?" At last a rusk was granted me to sop in my tea, and I ate it with rapture. The next day he said, "Since you are so very hungry, Fräulein, you shall have for your dinner a tiny slice of"—now you will hardly believe me, my readers, but it is absolutely true—"a very tiny slice of raw ham." Think of refusing me boiled chicken and offering me raw ham! It is the one thing I have never understood in my clever doctor's treatment. But not even in my hungriest pangs could I contemplate the possibility of swallowing a mouthful. I cried with disappointment, and the doctor had a worse opinion of English fancifulness than ever. The rest of his treatment seemed to me beautifully reasonable. Perfect quiet, both for mind and body; the subduing of the heat of the body by outward applications of cold; and as few demands made as possible on the digestive function. Nature, as it were, patted on the back during her hard fight, and encouraged to win. And win she did. Of course I was very weak at first, but it was a natural and childlike weakness, not the exhaustion which so often follows fever. I craved for food as an infant craves; but I was only allowed to have what I could digest and assimilate. No projects were tried which could bring on a relapse, and so I made slow but steady progress.

I was not allowed to stand for what seemed a long time, but at last I was put on my feet, and soon discarded the aid of the good sisters who at first helped me in my promenade up and down the corridor. Then it was I found out that I was on the men's floor; and after a bit I made acquaintance with some of the navvies who were making a line of rail near the town, and who had come in as sufferers from various accidents. One was an Italian count who had a broken arm, and with him I used to pace up and down and discuss various problems respecting the Catholic faith, which he had thrown off as inconsistent with scientific discoveries, and the policy of the Government which had reduced him to earn his living by working with his hands as a day labourer.

As soon as I was able to be properly dressed and could walk without assistance I was permitted by my kind doctor to go with him on his rounds, and he would explain to me any interesting cases in the wards, and the method of treatment pursued. One day, as a great treat, I was invited into the operating theatre to see a man's foot amputated. Of course, it was because he knew me to be a trained nurse that I was made thus free of the hospital. And because I was accustomed to surgical work, and all its adjuncts, these sights, which, offhand, do not seem particularly suitable for a convalescing patient, did me no harm; but rather took off my thoughts from myself, which was an excellent thing. At the end of eight weeks I left the hospital perfectly well, though not very strong; and yet strong enough to go almost straight to the *Hebammen Schule*, and begin my work there, which involved getting up at 6 a.m. on January mornings.

And now, after nearly twenty years, when I look back at Ludwig's Spital, I see it through a halo of grateful memories. Dr. Teuffel and even Dr. Essig shine like stars on a dark night; and, as for good little *Schwester* Röslie, when sentimental maidens talk to me of the angelic calling of a nurse, and how sweet it must be to smooth the dying pillow, I think of her dear, dumpy, little figure, and how she nursed me back to life.

### Novelties for Nurses.

### THE LONDON SHOE COMPANY.

The London Shoe Company has recently opened a City warehouse at 123 and 125, Queen Victoria Street, E.C. From a visit we paid to this establishment a few days ago we formed the opinion that it is probably the largest establishment of the kind in the country, if not in the world. Here every kind and pattern and size of shoe may be procured without delay or difficulty. The shoes are manufactured by the London Shoe Company, which guarantees the quality of all the goods except the patent leathers, which no shoemaker, we believe, will warrant, because that would be an impossibility. The vastness of the establishment will be realised when we state that the top floor of the warehouse is entirely devoted to satin shoes, and there must be many thousands of pairs of this article in stock. So many of our readers are customers of the London Shoe Company that it is unnecessary to assure them of the excellent quality of the goods which this enterprising firm have now brought within the reach of everybody.

### SOUTHALL'S SPECIALITIES.

We have much pleasure in testifying to the excellence and usefulness of Messrs. Southall's specialities. They are so well known that little remains to be said about them, but we would especially draw attention to the accouchement sheets, which are almost indispensable, and once adopted would never be lost sight of by any nurse. They are hygienically excellent and economical in use.

### Appointments.

Dover Hospital.—The post of Honorary Lady Superintendent at the Dover Hospital has been filled by Miss Helena Atthill, who was trained at the Middlesex Hospital and the Rotunda Hospital, Dublin. She held the post of night superintendent at the Staffordshire General Infirmary, and that of sister at the Monsall Hospital, Manchester.

Dundee Royal Infirmary.—Miss Jessie M. Duff, who was trained at the Charing Cross Hospital, has been appointed Matron of the above infirmary.

Keighley and Bingley Fever Hospital.—Miss A. Findlay has been appointed Matron of this institution. She was trained at the Liverpool Workhouse Infirmary, was charge nurse at the Liverpool Stanley Hospital for thirteen months; later she was parish nurse at Stone in Staffordshire for two years. She then held the post of charge nurse at the Mill Road Infirmary, Liverpool, from November, 1893, to April, 1895, leaving to become head nurse at the Keighley Union Infirmary to the present time. Miss Findlay shows excellent testimonials throughout her career.

### Nightingale Nurses.

Miss Gordon, the matron of the hospital and superintendent of the school, reports that another year had passed of quiet, steady work, that the probationers had attended the usual courses of lectures, and that Miss Crossland, the home sister, had been most indefatigable in helping and encouraging the probationers with their studies, and in looking after their comforts in the home. She mentions that the opening of two wards, Florence and Beatrice, which had long been closed, had made it necessary to increase the number of probationers from thirty-nine to forty-five, and that as there was no accommodation in the Nightingale Home for any addition to the number of occupants, rooms had been provided for this additional number of probationers in the house within the hospital, which had been recently adapted for the use of some of the sisters not attached to wards and nurses—formerly the apothecary's house—and that these probationers though sleeping out of the Nightingale Home would continue to be subject to the rules of the home, and be under the supervision of the home sister.

The report of the Fund further says that, owing to the constant changing of probationers, their varied previous education, and the short time of their residence in the home (one year only being the period of the probationary course), in comparison with the amount of practical information which the teacher would desire to impart to them, it has been increasingly difficult for the home sister to conduct the teaching in as regular and systematic a manner as would be satisfactory to her. The special or lady probationers attend, as above mentioned, three courses of twelve lectures by members of the hospital staff. One lecture is given in the week. The home sister attends these lectures with the matron, and herself takes notes, as the probationers also are expected to do. The probationers (for convenience of ward attendance) are divided into two classes, and the home sister takes each class once a week from eleven till half-past twelve a.m., goes over her notes with them, explaining anything which they do not understand, and each probationer afterwards makes a clear copy of the notes. During the three months in which there are no lectures, July, August, and September, she gives class instruction on bandaging, weights and measures, abbreviations, reading of bed tickets, and any other subjects the probationers may desire.

The nurse probationers are divided into three classes, also for the convenience of ward attendance, and the home sister takes each class once a week, from eleven to twelve in the morning. The subjects of instruction are elementary anatomy and physiology, in addition to those above mentioned, and any other subjects which may be of use in their practical work in the wards. After each class each probationer writes out her notes for correction by the home sister.

Every week one probationer is called upon to write a diary of her work in her ward, which is submitted to the home sister, and by her discussed with the writer. The home sister has endeavoured to keep up a constant training in habits of puctuality, neatness, cleanliness, quietness—to maintain a good tone in the "home"—to instil into the probationers that to become good practical useful nurses, they must cultivate every womanly quality. She has tried to let them feel that they may come to her at any time to ask questions, which she would answer to the best of her ability, or, if unable, she would direct the inquirers to the source where the information could be found.

This very brief summary may afford some idea of the manner in which Miss Crossland has endeavoured to fulfil the duties of home sister and to carry out the principles of moral training, which Miss Nightingale has constantly inculcated, as often referred to in these reports, as well as to afford to the matron, as the head of the school, that practical and loyal assistance without which a large hospital training school for nurses could never be efficiently conducted.

### Everybody's Opinion.

[Correspondence on all subjects is invited, but we cannot in any way be responsible for the opinions expressed by our correspondents. No communications can be entertained if the name and address of the correspondent is not given, or unless one side of the paper only be written on.]

### THE TRUTH OF THE MATTER.

"X Y Z" writes: I was delighted when I saw the above heading in The Hospital a few weeks back, for it expressed exactly what I have felt and known to be the truth, or at least part of it, ever since the three years' question has been on the "tapis," and I hope you will allow me to express my opinion on the subject in your paper, as I know that you do not hold yourself responsible for the opinions of the correspondents' column. Now for the first part of the truth of the matter. It has always struck me most forcibly from the beginning of the three-year system that its advocates were either matrons who thought it a good plan for keeping a hold on nurses who frequently left the hospital before their time was up, and yet who easily got employment on leaving, and that they (the—in the hospital—all-powerful lady superintendents) were absolutely powerless to punish or injure; or by their fellow nurses who, having done their three, and, perhaps, twelve, years in hospital, were still unsuccessful in their career, and were embittered to see how some young probationer they had taught to make a bed or apply a fomentation after a couple of years leave the hospital (either through her health failing her, or through a death or illness in the family, which compelled her to leave certificateless), succeed in making a good connection for herself. And just when she had grown so useful in the hospital! (It's lies) But what is your definition of a fully-trained nurse? What constitutes one? and what standard do you aim at? Again, how can you lay down a fixed period of time and qualify and disqualify a nurse, even if there were a universal curriculum? The difficulties are enormous. 1. Differences of class, varying from the cultured woman down to the cottage girl. 2. Difference of age, commencing at 18, ending at 30 and even more. 3. Difference in size of training school, varying from 40 beds to 800. 4. Difference in posts to be held after termination of training. How can the simple, unsophisticated, uneducated village girl learn at a fixed period of time what the educated mind grasps, or the young girl of 20 what the maturer mind of 30 does? And again, how can the girl trained in a hospital of 40 beds see and acquire what the other has done in a hospital of 400? And what need has the girl who is willing to be a hospital drone or a village nurse to learn what the lady superintendent or sister of a large London hospital must know? It is not the three years that are at fault, but the chaotic system of training. It is that untrained women are received in hospitals simply as a means to an end, *i.e.*, to do the work, that is at the root of the evil; it is because probationers are let loose in the wards to pick up the art of nursing as best they can that produces the nurse whom the advocates of the three years' system so nobly wish to protect the public against. Have they succeeded? No! Their seven years' crusade leaves them where they have started; in fact, far worse, for the disgraceful goings on in the R.B.N.A. meetings have only made matters worse, and my belief is that, however much they may talk, they will not make the three years' system a law. What law is to prevent the public—nay, even doctors—to prefer being nursed by or employing a nurse who is bright, clever, full of tact and sympathy, so long as she knows how to make them comfortable and carry out the doctor's orders? Such a nurse will far sooner make herself popular and a good connection after only eighteen months or two years' training than the fully-trained one, who finishes her swabs to a turn and drapes a room in antiseptic white for even the opening of a carbuncle, and insists on having a clean towel to wring out a fomentation to be applied on a part where there isn't even a scratch of a pin for a wound. And what law is to prevent the matron of a hospital to give the post of "sister" to a nurse who has not had three years' training? Therefore, so long as the public will be nursed by nurses who have not had three years' training, and as long as doctors employ them, and as long as matrons will give sisters' posts to nurses who have not had three years' training, and as long as committees will give posts as matrons to nurses who have not had three years' training, so long will the three years' system prove a failure. A little more method in training probationers, and two standards—one for the educated and one for the uneducated woman—seems to me a more practical and just line to follow.

### ANSWERS TO CORRESPONDENTS.

Edward Staton, National Temperance Nurses' Co-operation, 8, Great Marylebone Street, W.—Your letter is unsupported by evidence, and therefore not one that can properly be published in its present form. We have frequently cautioned nurses of all classes againt entering the service of so-called co-operative institutions which are solely conducted with the object of making a profit for the individuals who run them. It is a misuse of the word co-operation to apply it to any institution which is not managed by a committee solely for the benefit of its members, who take all the profits, and are supplied at regular intervals with a financial statement duly audited by a public accountant. Any nurse who joins a so-called co-operation which does not fulfil these reasonable conditions has little cause to complain if the result causes them loss and disappointment.

### Asylum News and Nursing.

### A LETTER FROM SOUTH AFRICA.

An accident, the result of which might well have been more serious than it was, occurred not long ago at one of the Colonial asylums. A nurse, in returning from the doctor's office to the wards, was attacked by a male patient—a Hottentot—who struck her on the head with a large stone, and when she fell down, hit her with it again in the same place. This much was accomplished before anyone could reach her, although help was close at hand. The unfortunate nurse was put in a cab and taken to the hospital without a minute's delay; and, a capable surgeon being on the spot, the operation of trephining was performed at once, a square inch of bone being removed from the skull. It was quite successful, and the girl made a steady recovery. No doubt this accident, involving such a serious shock to the nerves, would have put an end to her career as a mental nurse (in which profession she had proved herself extremely capable); however, she has solved this problem, or is about to solve it, by getting married, the most satisfactory way for her of escaping the difficulty. She has also, of course, a claim on the Government.

The patient who injured her was known to be dangerous, and was on this occasion in charge of one of the gardeners. The latter having turned his back for a minute, the patient slipped away and followed the nurse, from no motive, apparently, but unreasoning rage. In the same irresponsible manner he had, some time previously, attacked a child. It is said that an order had been given that this patient was not to be taken out, and if so there was, of course, a neglect of duty somewhere. When a patient is a good worker, and has been quiet for a long time, there is a great temptation, even to a conscientious attendant, to become a little bit lax in watching him, or to allow him a little more liberty than he had immediately after his last outbreak. The moral is—*never* trust a dangerous patient, however long he may have been quiet, or however much he may seem to have improved; and if you are in sole charge of such a one, never take your eyes off him for an instant. This is a hard saying, but a necessary one.

One wing of the new asylum at Capetown has been already opened, the rest of the building being not yet ready. For this reason the accommodation for nurses is not at present all that might be desired, but no doubt everything will be of the best when it is completed. The old buildings were inconvenient in many ways, and ill-adapted for their purpose, besides being much too small. They were originally, I believe, merely farm buildings, afterwards used as a reformatory for boys. The entrance was bad, and, for a new-comer, very difficult to find.

The situation, on the summit of a hill, overlooking a wide stretch of lovely country, is in every respect all that could be desired for an asylum, and there are delightful walks and drives in the neighbourhood. The suburbs of Capetown can hardly be excelled in beauty by those of any other town in the world.

### For Reading to the Sick.

EFFORTS.

**Motto.**

Man must toil for good, or he shall toil for ill.
—*Lord Houghton.*

**Verses.**

Oft in a dark and lonely place,
I hush my hasten'd breath,
To hear the comfortable words
Thy loving Spirit saith;
And feel my safety in Thy hand
From every kind of death.

O, there is nothing in the world
To weigh against Thy will!
E'en the dark times I dread the most
Thy Covenant fulfil;
And when the pleasant morning dawns
I find Thee with me still.
—*Hymns and Meditations.*

Faith's meanest deed more favour bears,
Where hearts and wills are weigh'd,
Than brightest transports, choicest prayers,
Which bloom their hour and fade.
—*Newman.*

God doth not need
Either man's works, or his own gifts; who best
Bear His mild joke, they serve Him best. His state
Is kingly; thousands at His bidding speed,
And post o'er land and ocean without rest;—
They also serve who only stand and wait.
—*Milton.*

We shall marvel why we grudged
Our labour here, and idly judged
Of Heaven. —*Browning.*

**Reading.**

A subject of great trial to all sick people is, What they ought to do? what efforts they ought to make? They dread much more the moral and spiritual effects of overtasking strength than any mere bodily pain. They know that with every fresh exertion the weary languor increases, the suffering of which no words can tell; that it is not only suffering of body, but of soul which is involved in it: the languor hindering you from prayer, until the spiritual sight becomes dim; the nervousness, too, causing such irritability that the day is spent in struggle, in fear lest you should grieve your Lord and Master. The incapacity for all common duties, causing the fear that you are sinfully self-indulgent; the discontent consequent on this; the fear that others will misunderstand you, and think that is indolence, which is to you great suffering; the endless length that your future life of pain and weariness appears; the greatness of your sins; the intense feeling of the want of sympathy in those around. The burden of weakness which you are bearing makes it seem to you as if all cares and trials centred in you, and that all must be borne and done in this moment of unutterable incapacity. Then you feel that you shall never come out of it, and that seems in itself a wearisome weight of woe. Be not discouraged; you must not repine about it; you cannot help feeling the pain and suffering; you may have a grateful, thankful heart in spite of it all, and be very conscious of His presence and help. What the efforts are which will prove assistance in bearing the burden of sickness must of necessity differ in each case; each according to their several measures of weakness or ability. It is better to try what can be done, and be sure there is One Whom, if all others misunderstand, will understand it, One who can truly direct you what to do at all times.—From *Manual of Sickness: Its Trials and Blessings.*

### Minor Appointments.

Merthyr Tydfil Union Infirmary.—Miss Florence Brooks has been appointed Head Nurse at the above infirmary. She was trained at the Birmingham Workhouse Infirmary, where she held the post of charge nurse, and later joined the staff of the Leicester Institution for Trained Nurses, which she leaves to go to Merthyr Tydfil.

Cheshire County Asylum, Macclesfield.—Miss L. Haslewood has been appointed Head Nurse of this asylum. She was trained at the Marylebone Infirmary, where she also held the post of sister, and later that of night superintendent at the Brompton Hospital.

The Colonial Nursing Association.—Nurse E. Bothwell, who was trained at the Paisley Infirmary, and later at the Fever Hospital, and Nurse A. M. Cunnington, who was trained at the Nottingham Children's Hospital, the Brownlow Hill Infirmary, Liverpool, and the Jersey Fever Hospital, have both been selected for service in connection with this association.

### Notes and Queries.

**Queries.**

(125) *Buxton.*—Can you tell me of a convalescent home at Buxton?—*T. P.*

(126) *Colonial Nursing.*—Please give me information as to the New Colonial Nursing Association.—*Nurse C.*

(127) *Surgical.*—Can you tell me what date the examination for F.R.C.S. is held, and where?—*Sister.*

(128) *Children's Nurse.*—Can you tell me of an institution for training ladies as children's nurses?—*F. T.*

(129) *Dispensing.*—How can I learn dispensing without attending classes?—*A. C.*

(130) *Nursing Home.*—Is it necessary to get permission to open the above?—*Nurse M.*

(131) *Training.*—Where can nurses be trained at 38 years of age?—*Miss S.*

(132) *Mildmay Home.*—To whom ought I to apply respecting this home?—*Nurse K.*

(133) *Asylum Nursing.*—Is there a paper which publishes vacancies for nurses at private and public asylums?—*Kate.*

(134) *Anatomy.*—Kindly tell me of a simple book on anatomy.—*E.*

(135) *Health.*—Please give advice as to training as a hygiene lecturer.—*T. S.*

(136) *Midwifery.*—Where can a nurse who has had six months' training at a London hospital, and is a certified midwife, find employment?—*E. H.*

(137) *Book.*—Is there a book "Diseases and their Commencement"?—*Nurse S.*

(138) *Emigration.*—Where can I get information respecting nursing or asylum appointments in the Colonies?—*C. W.*

**Answers.**

(125) *Buxton (T. P.).*—Write to the Hon. Secretary, Hartington House, Buxton, and to the Devonshire Hospital. Why not apply to The Hospital Convalescent Fund, 428, Strand, for help?

(126) *Colonial Nursing (Nurse C.).*—Write to the Hon. Secretary of the Imperial Institute.

(127) *Surgical (Sister).*—Which examination? Both are held at the Examination Hall. Write to Mr. F. Hallett, Examination Hall, Victoria Embankment, W.C.

(128) *Children's Nurse (F. T.).*—Write to the Norland Institute, 19, Holland Park Road, or to the Moseley Hall Convalescent Home for Children, Worcestershire.

(129) *Dispensing (A. C.).*—You might study from books up to a certain point, and get a local chemist to teach you practical pharmacy. A useful chapter on this subject is to be found in Burdett's "Hospital Annual for 1894." We fear you could never expect to gain the qualification you require without proper practical teaching.

(130) *Nursing Home (Nurse M.).*—You cannot employ a habitation taken as a dwelling-house for any trade. Read carefully the wording of your lease or agreement.

(131) *Training (Miss S.).*—A list of hospitals and ages at which nurses are taken for training is given in "How to Become a Nurse" (Scientific Press). A paying probationer only, we believe, would be received at such an age. Please observe notice heading this column.

(132) *Mildmay Home (Nurse K.).*—To the Lady Superintendent, 9, Newington Green.

(133) *Asylum Nursing (Kate).*—Vacancies occasionally appear in all papers with advertisement columns, but we believe there is no so constant medium as our own columns.

(134) *Anatomy (E.).*—"Elementary Anatomy and Surgery for Nurses," by W. McAdam Eccles (Scientific Press, 428, Strand, W.C.).

(135) *Health (T. S.).*—Write to Miss Lamport, 52, St. John's Wood Road, who will be able to help you with excellent advice.

(136) *Midwifery (E. H.).*—You could obtain district nursing or workhouse nursing with your qualifications.

(137) *Book (Nurse S.).*—"Diseases and their Commencement," lectures to trained nurses by D. Hood, M.D. (J. and A. Churchill).

(138) *Emigration (C. W.).*—For nursing write to the Colonial Nursing Association, Imperial Institute. Watch Colonial papers or advertise and inform your matron of your wants.

## Figures and Tables

**Fig. 32. Fig. 33. f1:**
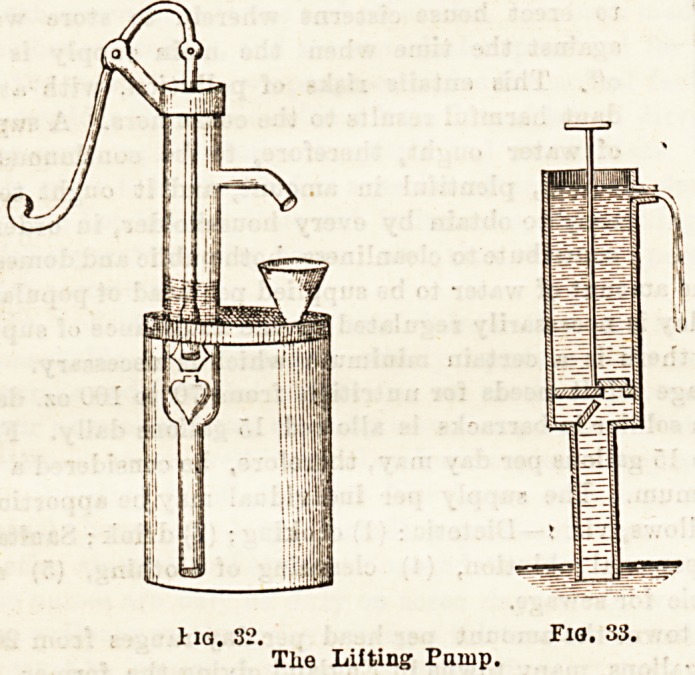


**Fig. 34. f2:**
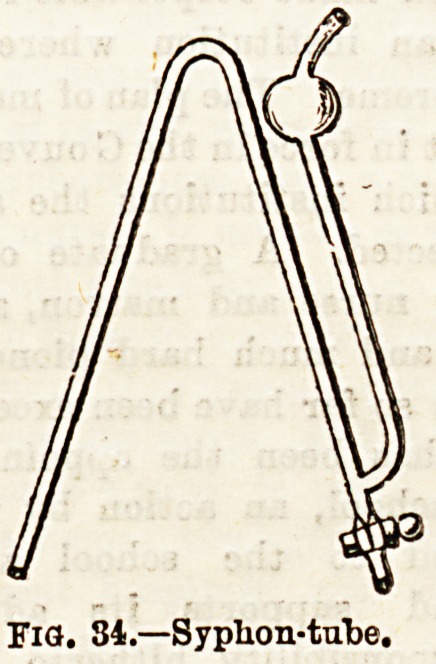


**Fig. 35. f3:**
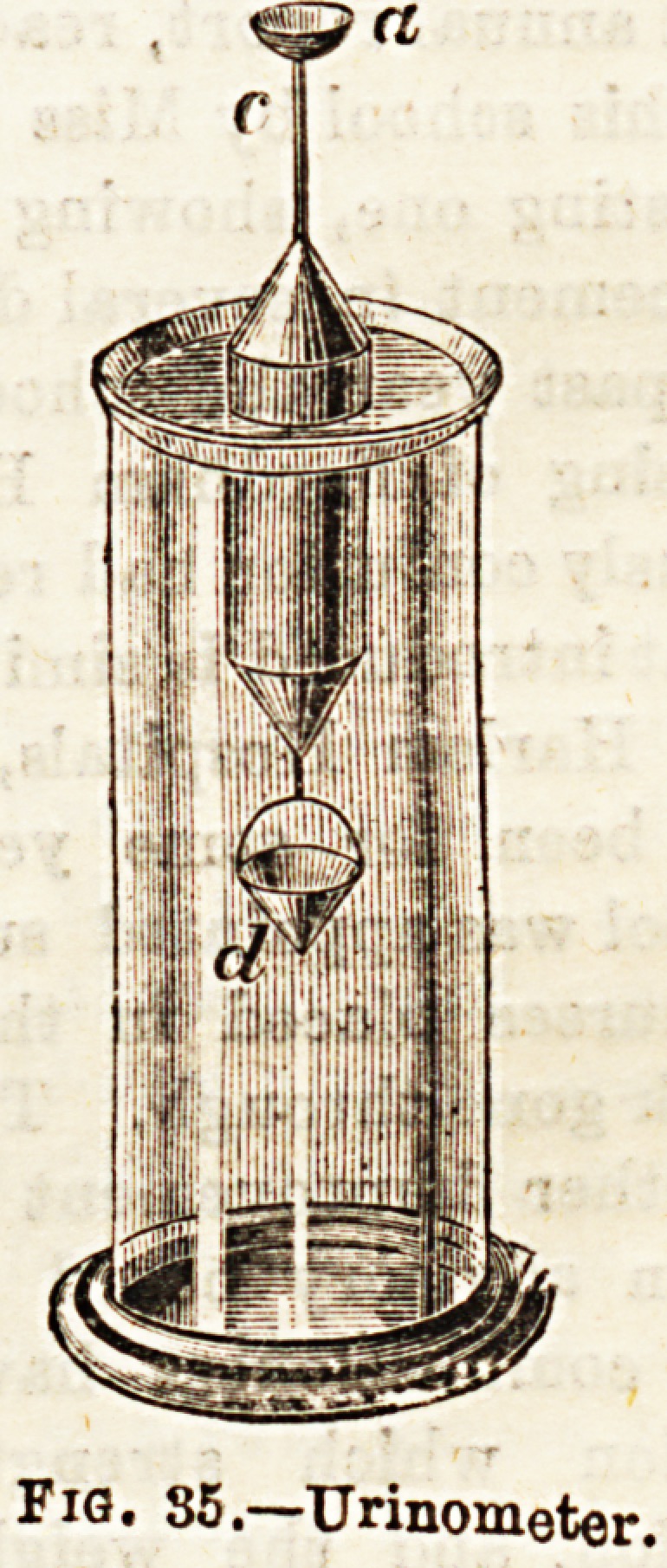


**Fig. 36. f4:**